# Nonrestorative sleep is associated with somatic and depressive symptoms in Japanese junior high school students

**DOI:** 10.1186/s40101-025-00412-8

**Published:** 2025-11-11

**Authors:** Yusuke Nakayama

**Affiliations:** Nakayama Pediatric Clinic, 1-23 Motohara-Machi, Nagasaki-City, Nagasaki, 852-8133 Japan

**Keywords:** Chronotype, Depression, Insomnia, Nonrestorative sleep, Restless legs syndrome, Restorative sleep, Sleep duration

## Abstract

**Study objectives:**

Nonrestorative sleep (NRS) has been identified as a potential risk factor for physical and mental well-being in adults, but limited research exists for children and adolescents. This study aimed to clarify the factors associated with NRS in Japanese junior high school students.

**Methods:**

The participants were 529 Japanese junior high school students in grades 7 through 9. Participants were asked to respond to Google Forms, and responses were obtained from 392 students. Sleep habits, history of coronavirus disease (COVID-19), physical symptoms, social isolation, and the presence of the symptoms of restless legs syndrome (RLS) were identified. NRS, insomnia symptoms, and depressive symptoms were evaluated using the Restorative Sleep Questionnaire (RSQ), Athens Insomnia Scale (AIS), and Patient Health Questionnaire (PHQ)-9, respectively. The cut-off value for NRS determination by the RSQ score was the mean of the scores that maximized the sensitivity and specificity sum for detecting participants with AIS and PHQ-9 scores of ≥ 6 and ≥ 5, respectively. NRS-associated sleep parameters and those associated with depressive symptoms were evaluated using binominal logistic regression analysis. Multinomial logistic regression (MLR) analysis was used to confirm the reproducibility of the binomial logistic regression analysis results with lower RSQ scores.

**Results:**

The NRS group comprised 40.1% of participants and exhibited a higher prevalence of physical and depressive symptoms compared to those with restorative sleep. Logistic regression analysis, adjusted for sex, grade, and COVID-19 history, revealed the following odds ratios (95% confidence interval) for NRS: average total sleep time < 7 h 2.44 (1.16–4.33), AIS ≥ 6 2.74 (1.51–4.95), evening chronotype 2.58 (1.49–4.47), and RLS symptoms 2.21 (1.21–4.03). The same results were obtained using MLR as those obtained via binomial logistic regression analysis. Logistic regression analysis for depressive symptoms revealed that NRS displayed the highest odds ratio (95% CI) of 3.16 (1.90–5.27) among the sleep-related variables.

**Conclusions:**

These findings suggest that NRS in Japanese junior high school students is associated with physical and mental health issues. Intervention and longitudinal studies are warranted to address NRS-associated sleep–wake problems in this age group.

**Supplementary Information:**

The online version contains supplementary material available at 10.1186/s40101-025-00412-8.

## Background

Nonrestorative sleep (NRS), the inability to feel fully rested and recover from sleep upon awakening, has been shown to be associated with physical and mental health problems in adults. A 2019 meta-analysis found that NRS increases the risk of all-cause mortality and death from cardiovascular disease [1]. In addition, NRS increases the risk of mortality among adults aged 40–64 years, among short sleepers, and among those aged 65 years and older [2]. Furthermore, NRS is associated with the development of metabolic syndrome, hypertension, and type 2 diabetes [3, 4], as well as the appearance of depressive symptoms [5].

NRS is a frequent sleep problem in adolescents as well as adults, occurring almost daily in 17.7% and often in 43.0% of adolescent students in the USA [6]. In a survey of adolescent students in China, when asked to identify which level of sleep restfulness, from 0 (no sleep rest at all) to 10 (fully rested), they reported that 4.5% scored 0 and had no sleep restfulness at all, with a mean (standard deviation) of 4.5 (2.30) [7]. Few studies have focused on NRS in adolescents, and, to the best of our knowledge, no studies on Japanese adolescents have been reported.

Although NRS is caused by a variety of sleep–wake problems, insomnia should also be considered a contributing factor to NRS in adolescents. Insomnia is diagnosed when there is difficulty falling asleep, staying asleep, or waking early in the morning, even with adequate sleep opportunities, and when there are daytime problems related to nighttime sleep difficulties. The prevalence of insomnia in adolescents is nearly 20% [8–10], and the prevalence of insomnia symptoms is even higher [11]. Insomnia symptoms that begin in childhood or adolescence often persist into adulthood [12], and need to be addressed appropriately.

Restless legs syndrome (RLS) should be distinguished from insomnia, as it is a sleep–wake disorder that presents with insomnia-like symptoms. Because RLS symptoms often emerge at bedtime and in the first half of sleep, RLS symptoms can cause difficulty initiating and maintaining sleep [13]. It is also evident that a substantial number of RLS cases have been inadvertently included in the population conventionally classified as having insomnia, particularly during efforts to identify disease susceptibility genes for insomnia [14]. The prevalence of RLS in children and adolescents is 1–6% [15–21], and it is important to consider the possibility of this syndrome when identifying insomnia symptoms.

The prevalence of RLS increased after the coronavirus disease (COVID-19) pandemic, and it is necessary to distinguish RLS from insomnia, especially as a potential sequelae of long COVID. The incidence and prevalence of RLS have increased several-fold, and more severe cases have also increased [22–24]. The affinity of dopaminergic neurons for severe acute respiratory syndrome coronavirus 2 has been shown to be higher than that of other neurons [25], which may contribute to the increase in RLS. Therefore, it is necessary to examine whether RLS in children and adolescents has increased after the pandemic. Given that COVID-19 infection affects not only RLS but also the broader aspects of sleep and circadian rhythms [26], it is essential to consider a history of COVID-19 infection in post-pandemic research, including studies on NRS.

This study aimed to determine the status of mental and physical health and sleep problems among Japanese junior high school students in 2024 after the spread of COVID-19. We confirmed sleep habits, insomnia symptoms, sleep restlessness, and RLS. In addition to anxiety and depressive symptoms, loneliness and isolation were identified as possible psychological problems. Fatiguability, loss of appetite, headache, and abdominal pain were identified as possible physical symptoms. In this study, we focused on NRS and examined and reported sleep–wake problems related to NRS to achieve better sleep health.

## Methods

### Participants, study design, and ethical considerations

All students (*N* = 529) enrolled in a junior high school in Nagasaki City in April 2024 were invited to participate in the study. We distributed an information disclosure document on the “Survey on Sleep Habits and Health Problems” to parents and ensured that parents and students had an opportunity to refuse participation in the study and use of the data. A total of 528 students were asked to respond to the survey, with the exception of one student whose parents requested not to participate in the study. The tablets distributed to all students in the study were used to collect data by requesting responses from Google Forms. All participants were given a randomly generated research ID, and their names and classes were excluded from data analysis. This study was approved by the Nagasaki City Medical Association Ethics Review Committee (approval no. 2023–5).

### Measures

#### Sociodemographic information

In this study, sex, grade, time of last use of electronic devices, past history of COVID-19, history of COVID-19 vaccination, physical and mental symptoms, insomnia symptoms, and sleep habits were investigated. The frequency of physical symptoms such as headache, abdominal pain, decreased appetite, and fatiguability was divided into the following four categories: "never,” "1 day a week,” "2–4 days a week,” and "more than 5 days a week.” Other items were checked using the following questionnaire.

#### Sleep habits

Sleep habits were assessed using the following seven items, and social jetlag (SJL) was evaluated[27]: 1. Last awakening time on weekdays, 2. Time of falling asleep on weekdays, 3. Last awakening time on weekends, 4. Time of falling asleep on weekends, 5. Total sleep time on weekdays (TST weekday), 6. Total sleep time on weekends (TST weekend), 7. Ideal total sleep time (ideal TST). From the answers to the above items, we calculated the average sleep time per week (average TST), assuming 5 weekdays and 2 weekends.

$$\text{sleep debt index }=\text{ideal TST }-\text{average TST}.$$

$$\text{average TST }= \left(\text{TST weekday }\times 5 +\text{TST weekend }\times 2\right) / 7\text{ days}.$$

Weekly sleep loss (wSL) was calculated as follows if the average TST was longer than the TST weekday: wSL = (average TST – TST weekday) × 5. In the other cases, wSL was calculated as follows: wSL = (average TST – TST weekend) × 2. wSLs were categorized into three groups: < 1 h, 1–2 h, and ≥ 2 h. SJL was calculated from mid-sleep time (MST). Relative SJL was calculated by subtracting the weekday MST from the weekend MST, and the absolute value of relative SJL was calculated.

Weekends MST was used to assess the chronotype of each individual. The MST of the participants was divided into three groups, approximately equal in number, and categorized as morning, intermediate, and evening types, starting with the early group. However, if the TST weekend was longer than the average TST, the MST on the weekends was corrected as follows: corrected MST weekend = MST weekend – (TST weekend – average TST)/2.

#### Restorative sleep questionnaire

The Japanese version of the Restorative Sleep Questionnaire is a 9-item self-administered questionnaire that measures the sense of restfulness obtained from sleeping during the past week [28, 29]. Each item is scored from 1 to 5, and the average score of the nine items is converted to a score from 0 to 100. The lower the score, the lower is the perceived recovery. Based on reports from Japanese participants, a score of less than 45.8 was considered to be NRS [28].

#### Athens insomnia scale

The Japanese version of the Athens Insomnia Scale (AIS) is an 8-item self-administered questionnaire that measures insomnia severity [30, 31]. It asks for responses to insomnia symptoms and sleep-related daytime problems in the past month. Each item is scored from 0 to 3, and the total score ranges from 0 to 24, with higher scores indicating greater severity of insomnia. The cutoff point between normal participants and those with insomnia is 6 points [30]. The severity of insomnia is determined as follows: 6–9 points is mild, 10–15 points is moderate, and 16–24 points is severe [32].

#### Restless legs syndrome single question

The presence or absence of RLS symptoms was confirmed by the following single question: when you try to relax in the evening or sleep at night, do you ever have unpleasant, restless feelings in your legs that can be relieved by walking or movement [33]? Those who answered “not at all” for the past 2 weeks were considered the non-RLS group, while the others were considered the RLS group. Furthermore, we divided the RLS group into two groups: those whose symptoms appeared 1 day per week and those whose symptoms appeared 2 or more days per week during the past 2 weeks.

#### Patient health questionnaire-9

The Japanese version of the Patient Health Questionnaire-9 (PHQ-9) is a 9-item self-administered questionnaire that measures depressive symptoms [34, 35]. Participants answer questions about their symptoms of depression over the past 2 weeks; each item is scored from 0 to 3, and the total score ranges from 0 to 27, with higher scores indicating greater severity of depression. A score of 5 or more indicates the presence of depression [35].

#### Generalized anxiety disorder-7

The Japanese version of the Generalized Anxiety Disorder-7 is a 7-item self-administered questionnaire that measures anxiety disorders [36]. The questionnaire asks about anxiety disorders in the past 2 weeks. Each item is scored from 0 to 3, with total scores ranging from 0 to 21, with higher scores indicating greater severity of anxiety disorder. A score of 5 or more indicates the presence of an anxiety disorder [36].

#### Three-item loneliness scale

The Japanese version of the Three-Item Loneliness Scale is a 3-item self-administered questionnaire that measures loneliness [37, 38]. The survey was designed to be easily answered, assuming a large-scale survey using telephones or other means. Each item is scored from 1 to 3, and the total score ranges from 3 to 9, with higher scores indicating a stronger sense of loneliness.

#### Lubben social network scale

The abbreviated Japanese version of the Lubben Social Network Scale is a 6-item self-administered questionnaire that measures social networks [39, 40]. Each item is scored from 0 to 5 on the family and friends network, and the total score ranges from 0 to 30, with higher scores indicating a larger social network.

### Statistical analysis

Data were analyzed using EZR version 1.68 [41] based on R ver. 4.3.1 (https://www.r-project.org/). Normality was evaluated using the Shapiro–Wilk test. Continuous variables for which normality was confirmed are expressed as mean (standard deviation), and continuous variables for which normality was not observed are expressed as median (interquartile range [IQR]). For comparisons between two groups of continuous variables, an uncorrelated *t*-test was used for normally distributed variables, and the Mann–Whitney *U* test was used for non-normally distributed variables. For comparisons between three or more groups of continuous variables, one-way analysis of variance was used for variables that showed normality, the Kruskal–Wallis test was used for variables that showed non-normality, and the Bonferroni method was used for multiple comparisons. Fisher’s exact probability test was used to test the independence of the nominal variables. The significance level was set at *p* < 0.05.

Although the NRS-related RSQ score cut-off value in Japanese adults is reportedly 45.8 [28], that in adolescents has not yet been reported. In this study, the NRS-related RSQ score cut-off value was determined in this age group based on the insomnia- and depressive symptom-associated RSQ scores. The receiver operating characteristic (ROC) curves of the RSQ scores for AIS and PHQ-9 scores of ≥ 6 and ≥ 5 were used to determine the RSQ score that maximized the sensitivity and specificity sum, respectively, using the mean of the two as the cut-off value in this study. The NRS group was defined as that below the cut-off value. The area under the ROC curve (AUC) and its 95% confidence interval (CI) were calculated.

Logistic regression analysis with NRS as the dependent variable was adjusted for sex, grade, and past history of COVID-19, and the following sleep-related variables were entered as independent variables: average TST, AIS score, chronotype, and RLS symptom. The average TST was categorized into four groups: < 7 h, 7–8 h, 8–9 h (ref), and ≥ 9 h. The AIS scores were categorized into two groups: < 6 (ref) and ≥ 6. RLS symptoms were categorized into two groups: those who had symptoms less than 1 day per week, including the non-RLS group, and those who had symptoms 2 or more days per week, with the non-RLS group and those who had symptoms less than 1 day per week as the reference group. In the multivariable analysis, a history of COVID-19 was included as one of the adjustment factors to account for the global increase in RLS prevalence associated with the pandemic and the impact of post COVID-19 conditions. Multinomial logistic regression (MLR) analysis was used to confirm the reproducibility of the results obtained by binomial logistic regression analysis with lower RSQ scores, i.e., with higher non-restorative sleep levels. MLR was performed by dividing the RSQ score into three groups in order of equal number of scores, and the odds ratio was calculated with the group exhibiting the highest score as the reference. Logistic regression analysis with a PHQ-9 score of ≥ 5 as the dependent variable was also performed by adding the NRS group with the RS group as the reference to the independent variable in the NRS analysis.

## Results

In this survey, 528 students were asked to respond to the questionnaire, and 476 responses were obtained, excluding 52 students who were absent owing to illness or long-term absence. Responses with missing values that would have affected the analysis were excluded, and 392 participants (185 boys and 207 girls) were included in the analysis. As of April 2024, half of the participants had completed the COVID-19 vaccination at least once, and 3/4 of the participants had been affected by COVID-19 at least once (Table 1).

**Table 1:** Demographic characteristics

n	392
Grade	
seventh, n (%)	122 (31.1)
eighth, n (%)	143 (36.5)
ninth, n (%)	127 (32.4)
Electronic devices user, n (%)	257 (65.6)
Last electronic devices usage time	257 (65.6)
< 21:00, n (%)	59 (15.1)
21:00–22:00, n (%)	76 (19.4)
22:00–23:00, n (%)	128 (32.7)
≥ 23:00, n (%)	129 (32.9)
Past history of COVID-19, n (%)	296 (75.5)
COVID-19 unvaccinated person, n (%)	195 (49.7)

*COVID-19* coronavirus disease

Of the participants, 64.0% were aware of fatiguability as a physical symptom, and 36.7% complained of fatigue more than twice a week. A PHQ-9 score of ≥ 5 was reported by 28.6% of the participants and was more common among females. Although the survey was conducted just after the entrance of seventh-grade junior high school students, more seventh-grade students reported feeling anxious, lonely, and lacking a social network compared to other grades (Tables S1 and S2).

Sleep duration decreased in the older grades, and students tended to be nocturnal. The median (IQR) average sleep duration was 8.00 (7.29, 8.57) h, and 48.2% of the participants slept for less than 8 h, although the recommended sleep duration for junior high school students is 8–10 h. An AIS score ≥ 6 was reported by 16.8% of the participants. RLS symptoms were observed in 15.8% of the participants, and 7.7% of the participants had symptoms more frequently than 2 days a week (Tables S1 and S2).

The histogram of RSQ score with bins of eight displayed non-normal distribution. Although not clearly bimodal, two peaks could be observed at 40–48 and 56–64. If an RSQ score cut-off value was to be set, it had to be somewhere in between these two peaks (Fig. S1). The RSQ score for a PHQ-9 of ≥ 5 with the maximum sensitivity and specificity sum was 53.1 as well as with a sensitivity, specificity, and AUC (95% CI) of 0.65, 0.71, and 0.73 (0.68–0.79), respectively (Fig. S2). In addition, that for an AIS of ≥ 6 was 50.0, with a sensitivity, specificity, and AUC (95% CI) of 0.65, 0.65, and 0.70 (0.63–0.77) (Fig. S3. The cut-off value for the RSQ score in this study was 51.6 of the mean of the two groups, and the participants were divided into NRS and RS groups.

The NRS group accounted for 40.1% of participants. Compared to the restorative sleep group, the NRS group was more likely to use electronic devices and more likely to use them late after 23:00. The NRS group was more likely to have physical symptoms, such as headache, abdominal pain, loss of appetite, and fatiguability, as well as depressive symptoms, while there was no association with anxiety, loneliness, or a lack of social networks (Table 2).

**Table 2:** Comparisons of demographic and psychosomatic symptoms between restorative sleep (RS) group and nonrestorative sleep (NRS) group

	RS group	NRS group	*P* value
n	235	157	
Male, n (%)	120 (51.1)	65 (41.4)	0.06
Grade			
seventh, n (%)	79 (33.6)	43 (27.4)	0.37
eighth, n (%)	85 (36.2)	58 (36.9)	
ninth, n (%)	71 (30.2)	56 (35.7)	
Electronic devices user, n (%)	140 (59.6)	117 (74.5)	0.002
Last electronic devices usage time			
< 21:00, n (%)	41 (17.4)	18 (11.5)	< 0.001
21:00–22:00, n (%)	54 (23.0)	22 (14.0)	
22:00–23:00, n (%)	83 (35.3)	45 (28.7)	
≥ 23:00, n (%)	57 (24.3)	72 (45.9)	
Headache, n (%)	61 (30.3)	73 (54.5)	< 0.001
≥ 2 days/week, n (%)	16 (6.8)	35 (22.3)	< 0.001
Abdominal pain, n (%)	62 (26.4)	75 (47.8)	< 0.001
≥ 2 days/week, n (%)	17 (7.2)	31 (19.7)	< 0.001
Appetite loss, n (%)	29 (12.3)	49 (31.2)	< 0.001
≥ 2 days/week, n (%)	12 (5.1)	19 (12.1)	0.02
Fatiguability, n (%)	118 (50.2)	133 (84.7)	< 0.001
≥ 2 days/week, n (%)	55 (23.4)	89 (56.7)	< 0.001
Past history of COVID-19, n (%)	176 (74.9)	120 (76.4)	0.81
COVID-19 unvaccinated person, n (%)	115 (48.9)	80 (51.0)	0.05
PHQ-9 score, median (IQR)	1.0 (0.0, 3.0)	4.0 (2.0, 8.0)	< 0.001
PHQ-9 score ≥ 5, n (%)	39 (16.6)	73 (46.5)	< 0.001
GAD-7 score, median (IQR)	1.0 (0.0, 4.0)	1.0 (0.0, 3.0)	0.94
GAD-7 score ≥ 5, n (%)	47 (20.0)	27 (17.2)	0.51
Loneliness scale score, median (IQR)	3.0 (3.0, 4.0)	3.0 (3.0, 4.0)	0.25
Loneliness scale score ≥ 6, n (%)	26 (11.1)	8 (5.1)	0.04
LSNS-6 score, median (IQR)	21.0 (16.0, 24.0)	21.0 (17.0, 26.0)	0.09
LSNS-6 score ≥ 12, n (%)	28 (11.9)	12 (7.6)	0.23

*IQR* Interquartile range, *GAD* Generalized Anxiety Disorder, *LSNS-6* Lubben Social Network Scale-6, *NRS* nonrestorative sleep, *PHQ* Patient Health Questionnaire, *RS* Restorative sleep, *RSQ* Restorative Sleep Questionnaire

Compared with the restorative sleep group, the NRS group had higher rates of AIS score ≥ 6, difficulty waking, and RLS symptoms. Sleep duration was shorter in the NRS group, reflecting the later time of sleep onset not only on weekdays but also on weekends. The chronotype tended to be evening-type, and sleep loss and SJL were prominent (Table 3).

**Table 3:** Comparisons of sleep characteristics between restorative sleep (RS) group and nonrestorative sleep (NRS) group

	RS group	NRS group	*P* value
n	235	157	
AIS score, median (IQR)	2.0 (1.0, 4.0)	3.0 (2.0, 6.0)	< 0.001
AIS score ≥ 6, n (%)	23 (9.8)	43 (27.4)	< 0.001
RSQ score, median (IQR)	68.8 (59.4, 78.1)	40.6 (31.3, 43.8)	< 0.001
Difficulty getting up, n (%)	33 (14.0)	59 (37.6)	< 0.001
RLS symptoms, n (%)	25 (10.6)	37 (23.6)	< 0.001
≥ 2 days/week, n (%)	10 (4.3)	20 (12.7)	0.003
Average sleep duration h, median (IQR)	8.00 (7.54, 8.57)	7.79 (7.14, 8.46)	0.009
< 7 h, n (%)	27 (11.5)	35 (22.3)	0.006
7–8 h, n (%)	72 (30.6)	55 (35.0)	
8–9 h, n (%)	104 (44.3)	47 (29.9)	
≥ 9 h, n (%)	32 (13.6)	20 (12.7)	
Ideal sleep duration h, median (IQR)	9.00 (8.00, 10.00)	9.00 (8.00, 10.00)	0.31
Weekday sleep habits			
Sleep duration h, median (IQR)	8.00 (7.00, 8.00)	7.50 (7.00, 8.00)	0.01
Falling asleep time, h:mm, median (IQR)	22:45 (22:00, 23:17)	23:00 (22:30, 23:30)	< 0.001
Last waking up time, h:mm, median (IQR)	6:30 (6:30, 7:00)	6:40 (6:30, 7:00)	0.80
Weekend sleep habits			
Sleep duration h, median (IQR)	8.75 (8.00, 10.00)	9.00 (8.00, 10.00)	0.58
Falling asleep time, h:mm, median (IQR)	23:00 (22:30, 23:30)	23:30 (23:00, 0:00)	< 0.001
Last waking up time, h:mm, median (IQR)	8:00 (7:00, 9:00)	8:30 (7:30, 9:30)	0.002
Corrected mid-sleep time, h:mm, median (IQR)	3:08 (2:38, 3:30)	3:25 (3:00, 4:09)	< 0.001
Chronotype			
Morning type	95 (40.4)	36 (22.9)	< 0.001
Intermediate type	82 (34.9)	49 (31.2)	
Evening type	58 (24.7)	72 (45.9)	
Weekly sleep loss, h, median (IQR)	1.43 (0.71, 2.86)	2.14 (1.43, 2.86)	0.04
< 1 h, n (%)	144 (61.3)	77 (49.0)	0.04
1–2 h, n (%)	61 (26.0)	57 (36.3)	
≥ 2 h, n (%)	30 (12.8)	23 (14.6)	
SJL, mm, median (IQR)	57.5 (30.0, 77.5)	75.0 (30.0, 112.5)	< 0.001

*AIS* Athens Insomnia Scale, *IQR* Interquartile range, *RLS* restless legs syndrome, *RSQ* Restorative Sleep Questionnaire, *SJL* social Jetlag

In the multivariate analysis with NRS as the dependent variable, an average TST < 7 h, an AIS score ≥ 6, evening chronotype, and RLS symptoms at least twice a week were identified as significant factors. In the MLR, the RSQ scores were divided into three groups, i.e., ≤ 50, 50–65.6, and > 65.6 (reference). The significant variables in the binominal logistic regression analysis also revealed proportional odds (Table 4).

**Table 4:** Logistic regression analysis for nonrestorative sleep

	Univariable analysis	Multivariable analysis*	MLR*
	Odds ratio (95% CI)	Odds ratio (95% CI)	Odds ratio (95% CI)
Average TST			
< 7 h	2.52 (1.36–4.68)	2.24 (1.16–4.33)	2.10 (1.16–3.84)
7–8 h	1.27 (0.75-–2.15)	1.49 (0.88–2.53)	1.28 (0.81–2.01)
8–9 h	1	1	1
≥ 9 h	0.86 (0.41–1.81)	1.28 (0.63–2.61)	0.78 (0.41–1.47)
AIS score			
< 6	1	1	1
≥ 6	3.25 (1.89–5.60)	2.74 (1.51–4.95)	2.76 (1.59–4.91)
Chronotype			
Morning type	1	1	1
Intermediate type	1.61 (0.90–2.87)	1.39 (0.80–2.40)	1.42 (0.89–2.26)
Evening type	3.42 (1.96–5.96)	2.58 (1.49–4.47)	2.45 (1.51–4.01)
RLS symptoms			
< 2 days/week or symptom-free	1	1	1
≥ 2 days/week	2.98 (1.71–5.19)	2.21 (1.21–4.03)	1.90 (1.09–3.38)

In the multinomial logistic regression (MLR), the RSQ scores were divided into three groups: ≤ 50, 50–65.6, and > 65.6 (reference). *Adjusted for sex, grade, and past history of COVID-19. CI: confidence interval, MLR: multinominal logistic regression, PHQ: Patient Health Questionnaire, RLS: restless legs syndrome

NRS was associated with higher PHQ-9 scores, and the PHQ-9 of ≥ 5 group displayed higher AIS scores, more short-sleepers, and more participants with RLS symptoms (Tables 2, S3, and S4). To examine the association between depressive symptoms and sleep-related factors including NRS, logistic regression analysis was conducted with a PHQ-9 of ≥ 5 as the dependent variable, and the following factors were significantly associated with depressive symptoms: mean sleep duration less than 7 h, AIS ≥ 6, RLS symptoms at least 2 days per week, and NRS (Table 5). As 112 students were involved in the PHQ-9 score ≥ 5 group, this analysis was corrected only for sex and grade, and not for COVID-19 status.

**Table 5:** Logistic regression analysis for PHQ-9 score ≥ 5

	Univariable analysis	Multivariable analysis^a^
	Odds ratio (95% CI)	Odds ratio (95% CI)
Average total sleep time		
< 7 h	2.99 (1.58–5.66)	2.27 (1.09–4.73)
7–8 h	1.91 (1.11–3.29)	1.64 (0.89–3.00)
8–9 h	1	1
≥ 9 h	1.16 (0.55–2.47)	0.97 (0.42–2.26)
Athens Insomnia Scale score		
< 6	1	1
≥ 6	4.27 (2.46–7.40)	3.01 (1.62–5.58)
Chronotype		
Morning type	1	1
Intermediate type	0.92 (0.53–1.61)	0.67 (0.36–1.25)
Evening type	1.56 (0.92–2.66)	0.79 (0.42–1.48)
RLS symptoms		
< 2 days/week or symptom-free	1	1
≥ 2 days/week	3.33 (1.91–5.82)	2.53 (1.36–4.72)
RSQ score		
Restorative sleep	1	1
Nonrestorative sleep	4.37 (2.74–6.95)	3.16 (1.90–5.27)

^a^Adjusted for sex and grade. *CI* confidence interval, *PHQ* Patient Health Questionnaire, *RLS* restless legs syndrome

## Discussion

Japanese junior high school students with fatiguability and depressive symptoms accounted for approximately 30% of the total number of students. NRS was also observed in approximately 40% of the students. The presence of NRS was significantly associated with mental and physical problems. Short sleep duration, insomnia symptoms, evening chronotype, and RLS symptoms were identified as sleep problems associated with NRS. To achieve sleep health for adolescents to lead healthy lives, longitudinal and intervention studies addressing NRS-related concerns would be required.

The NRS is associated with difficulty in waking. When adolescents wake up spontaneously in the morning and are able to get out of bed promptly, they often experience a sense of restorative sleep. If they have to repeatedly rely on an alarm or be repeatedly woken up by family members, their performance in the morning may decline, along with their likelihood of maintaining the habit of eating breakfast [42]. NRS is an easy-to-understand indicator that can be felt by adolescents themselves and their families. Intervention effects on specific sleep–wake problems related to NRS has to be examined.

In this study, the prevalence of symptomatic participants suspected of having RLS was higher than expected and was an important factor associated with NRS. Although the prevalence and incidence of RLS in adults are increasing with the spread of COVID-19 worldwide [22–24], to our knowledge, there are no reports of RLS in children and adolescents. RLS symptoms emerge during the night and late at night, which may cause not only insomnia but also the evening chronotype with late onset of sleep. The epidemiology and clinical findings of RLS in children and adolescents need to be re-examined.

This study identified a significant association between NRS and depressive symptoms. Logistic regression analysis, with a PHQ-9 of ≥ 5 as the dependent variable, indicated that NRS yielded the highest odds ratio among sleep-related factors. The link between depression, depressive symptoms, and sleep disturbances is well-established, even in adolescents [43–46]. NRS is likely to serve as a relatively simple indicator for identifying mental health problems in students of this age group.

This study had some limitations. First, the questionnaires used in this study were the same as those used with adults. There is a concern that adolescents might perceive and report NRS, insomnia symptoms, and depressive symptoms differently from adults, potentially leading to misunderstandings regarding the questionnaire contents. This discrepancy could result in a mismatch in the applicability of the scale, potentially causing either an overestimation or underestimation of the prevalence estimates. The valid response rate of 82.4% was low, which may have been due to the content of the questionnaire. For example, the Patient Health Questionnaire for Adolescents (PHQ-A) has been developed for the PHQ-9 for adolescent participants [47]. Although a validated Japanese version of the PHQ-A has not been published, a questionnaire for this age group should be utilized whenever possible, and sufficient consideration should be given when an appropriate questionnaire is not available. Second, it is important to acknowledge that the RSQ contains items that overlap with those in the PHQ-9, which may inevitably affect and enhance the correlation between the two questionnaires. The PHQ-9 is a questionnaire designed to assess the extent to which an individual has been affected by depressive symptoms over the preceding two weeks. In contrast, the RSQ is focused on symptoms experienced specifically upon waking. Consequently, it is assumed that responses to similar symptoms may not necessarily align. Nonetheless, it remains possible that the two questionnaires could exert mutual influence. Third, the present study could not include information on absenteeism, which corresponds to approximately 10% of the target population, because the responses were requested at school. There is a concern that some absent students may have sleep–wake problems. The inclusion of responses from absent students may have resulted in a higher number of students with problems. Fourth, there is a lack of comprehensive evaluation of the daily changes in sleep–wake problems associated with NRS. In addition to insomnia, RLS, and circadian rhythm sleep–wake disorder, NRS is also observed in long-sleepers and patients with hypersomnia, in whom the effects of sleep insufficiency are more likely to become apparent. Obstructive sleep apnea and periodic limb movement disorder, which cause poor sleep quality, also affect NRS. There is a need for a more comprehensive evaluation of sleep–wake problems because questionnaires alone have limitations.

## Conclusions

In conclusion, NRS is associated with physical and mental health problems among Japanese junior high school students. Although the NRS was evaluated using a questionnaire originally designed for adults, the future development of an assessment method tailored for adolescents is imperative. Factors such as short sleep duration, insomnia symptoms, evening chronotype, and RLS symptoms are associated with NRS. Moreover, NRS emerged as a significant sleep parameter in relation to depressive symptoms. In studies applying the NRS, accounting for individual sleep–wake characteristics and NRS-associated comorbid sleep–wake disorders is crucial. Future intervention and longitudinal studies using NRS as an index are warranted.

## Supplementary Information

Supplementary Material 1: Table S1. Sex differences of demographic characteristics, psychosomatic symptoms, and sleep–wake characteristics. Table S2. Grade differences demographic characteristics, psychosomatic symptoms, and sleep–wake characteristics. Table S3. Comparisons of demographic and psychosomatic symptoms between PHQ-9 score < 5 group and PHQ-9 score ≥ 5 group. Table S4. Comparisons of sleep characteristics between PHQ-9 score < 5 group and PHQ-9 score ≥ 5 group. Figure S1. Histogram of RSQ score. Figure S2. ROC curve of RSQ score for PHQ-9 ≥ 5. Figure S3. ROC curve of RSQ score for AIS ≥ 6. 

## Data Availability

The datasets used and/or analyzed during the current study are available from the corresponding author upon reasonable request.

## Abbreviations

AIS: Athens Insomnia Scale
COVID-19: Coronavirus disease
IQR: Interquartile range
MLR: Multinominal logistic regression
MST: Mid-Sleep Time
NRS: Nonrestorative sleep
PHQ-9: Patient Health Questionnaire-9
PHQ-A: Patient Health Questionnaire for Adolescents
RLS: Restless legs syndrome
SJL: Social jetlag
TST: Total sleep time
wSL: Weekly sleep loss
