# Supplementary material for: Nonrestorative sleep is associated with somatic and depressive symptoms in Japanese junior high school students
Source: J Physiol Anthropol. 2025 Nov 11;44:30. doi: 10.1186/s40101-025-00412-8 (PMC12607099; doi:10.1186/s40101-025-00412-8)
Supplement: Supplementary file 1 — Supplementary Material 1: Table S1. Sex differences of demographic characteristics, psychosomatic symptoms, and sleep–wake characteristics. Table S2. Grade differences demographic characteristics, psychosomatic symptoms, and sleep–wake characteristics. Table S3. Comparisons of demographic and psychosomatic symptoms between PHQ-9 score < 5 group and PHQ-9 score ≥ 5 group. Table S4. Comparisons of sleep characteristics between PHQ-9 score < 5 group and PHQ-9 score ≥ 5 group. Figure S1. Histogram of RSQ score. Figure S2. ROC curve of RSQ score for PHQ-9 ≥ 5. Figure S3. ROC curve of RSQ score for AIS ≥ 6. [file 40101_2025_412_MOESM1_ESM.docx]

**Supplemental Materials**

Table S1. Sex differences of demographic characteristics, psychosomatic symptoms, and sleep-wake characteristics

|  | Total | Female | Male | *P* value |
| --- | --- | --- | --- | --- |
| n | 392 | 207 | 185 | 0 |
| Grade |  |  |  |  |
| seventh, n (%) | 122 (31.1) | 70 (33.8) | 52 (28.1) | 0.45 |
| eighth, n (%) | 143 (36.5) | 71 (34.3) | 72 (38.9) |  |
| ninth, n (%) | 127 (32.4) | 66 (31.9) | 61 (33.0) |  |
| Electronic devices user, n (%) | 257 (65.6) | 133 (64.3) | 124 (67.0) | 0.60 |
| Last electronic devices usage time |  |  |  |  |
| < 21:00, n (%) | 59 (15.1) | 32 (15.5) | 27 (14.6) | 0.93 |
| 21:00–22:00, n (%) | 76 (19.4) | 42 (20.3) | 34 (18.4) |  |
| 22:00–23:00, n (%) | 128 (32.7) | 65 (31.4) | 63 (34.1) |  |
| ≥ 23:00, n (%) | 129 (32.9) | 68 (32.9) | 61 (33.0) |  |
| Headache, n (%) | 134 (40.0) | 77 (43.0) | 57 (36.5) | 0.26 |
| ≥ 2 days/week, n (%) | 51 (13.0) | 34 (16.4) | 17 (9.2) | 0.04 |
| Abdominal pain, n (%) | 137 (34.9) | 75 (36.2) | 62 (33.5) | 0.60 |
| ≥ 2 days/week, n (%) | 48 (12.2) | 28 (13.5) | 20 (10.8) | 0.44 |
| Appetite loss, n (%) | 78 (19.9) | 46 (22.2) | 32 (17.3) | 0.26 |
| ≥ 2 days/week, n (%) | 31 (7.9) | 23 (11.1) | 8 (4.3) | 0.02 |
| Fatiguability, n (%) | 251 (64.0) | 130 (62.8) | 121 (65.4) | 0.60 |
| ≥ 2 days/week, n (%) | 144 (36.7) | 83 (40.1) | 61 (33.0) | 0.17 |
| Past history of COVID-19, n (%) | 296 (75.5) | 155 (74.9) | 141 (76.2) | 0.81 |
| COVID-19 unvaccinated person, n (%) | 195 (49.7) | 109 (52.7) | 86 (46.5) | 0.08 |
| PHQ-9 score, median (IQR) | 2.0 (0.0, 5.00) | 2.00 (1.0, 6.0) | 2.0 (0.0, 4.0) | 0.009 |
| PHQ-9 score ≥ 5, n (%) | 112 (28.6) | 72 (34.8) | 40 (21.6) | 0.005 |
| GAD-7 score, median (IQR) | 1.0 (0.0, 3.00) | 1.0 (0.0, 3.0) | 1.0 (0.0, 4.0) | 0.20 |
| GAD-7 score ≥ 5, n (%) | 74 (18.9) | 34 (16.4) | 40 (21.6) | 0.20 |
| Loneliness scale score, median (IQR) | 3.0 (3.0, 4.0) | 3.0 (3.0, 4.0) | 3.0 (3.0, 4.0) | 0.97 |
| Loneliness scale score ≥ 6, n (%) | 34 (8.7) | 17 (8.2) | 17 (9.2) | 0.86 |
| LSNS-6 score, median (IQR) | 21.0 (16.0, 25.0) | 21.0 (16.5, 25.0) | 21.0 (16.0, 25.0) | 0.76 |
| LSNS-6 score ≥ 12, n (%) | 40 (10.2) | 22 (10.6) | 18 (9.7) | 0.87 |
| AIS score, median (IQR) | 3.0 (1.0, 5.0) | 3.0 (2.0, 5.0) | 2.0 (1.0, 4.0) | 0.04 |
| AIS score ≥ 6, n (%) | 66 (16.8) | 41 (19.8) | 25 (13.5) | 0.11 |
| Difficulty initiating sleep, n (%) | 185 (47.2) | 105 (50.7) | 80 (43.2) | 0.16 |
| Moderate–severe, n (%) | 39 (9.9) | 26 (12.6) | 13 (7.0) | 0.09 |
| Difficulty maintaining sleep, n (%) | 44 (11.2) | 23 (11.1) | 21 (11.4) | 1 |
| Moderate–severe, n (%) | 3 (0.8) | 2 (1.0) | 1 (0.5) | 1 |
| Early wakening, n (%) | 53 (13.5) | 27 (13.0) | 26 (14.1) | 0.77 |
| Moderate–severe, n (%) | 10 (2.6) | 3 (1.4) | 7 (3.8) | 0.20 |
| Poor sleep quality, n (%) | 150 (38.3) | 81 (39.1) | 69 (37.3) | 0.76 |
| Moderate–severe, n (%) | 14 (3.6) | 10 (4.8) | 4 (2.2) | 0.18 |
| Daytime sleepiness, n (%) | 285 (72.7) | 163 (78.7) | 122 (65.9) | 0.006 |
| Moderate–severe, n (%) | 68 (17.3) | 45 (21.7) | 23 (12.4) | 0.02 |
| RSQ score, median (IQR) | 56.3 (43.8, 71.9) | 53.1 (40.6, 68.8) | 62.5 (43.8, 75.0) | 0.004 |
| RSQ score < 51.6, n (%) | 119 (30.4) | 92 (44.4) | 65 (35.1) | 0.06 |
| Difficulty getting up, n (%) | 92 (23.5) | 52 (25.1) | 40 (21.6) | 0.47 |
| RLS symptoms, n (%) | 62 (15.8) | 32 (15.5) | 30 (16.2) | 0.89 |
| ≥ 2 days/week, n (%) | 30 (7.7) | 13 (6.3) | 17 (9.2) | 0.34 |
| Average sleep duration h, median (IQR) | 8.00 (7.29, 8.57) | 8.00 (7.29, 8.57) | 8.00 (7.29, 8.57) | 0.60 |
| < 7 hours, n (%) | 62 (15.8) | 36 (17.4) | 26 (14.1) | 0.68 |
| 7–8 hours, n (%) | 127 (32.4) | 63 (30.4) | 64 (34.6) |  |
| 8–9 hours, n (%) | 151 (38.5) | 82 (39.6) | 69 (37.3) |  |
| ≥ 9 hours, n (%) | 52 (13.3) | 26 (12.6) | 26 (14.1) |  |
| Ideal sleep duration h, median (IQR) | 9.00 (8.00, 10.00) | 9.00 (8.00, 10.00) | 9.00 (8.00, 10.00) | 0.42 |
| Weekday sleep habits |  |  |  |  |
| Sleep duration h, median (IQR) | 8.00 (7.00, 8.00) | 8.00 (7.00, 8.00) | 8.00 (7.00, 8.00) | 0.11 |
| < 7 hours, n (%) | 145 (37.0) | 88 (42.5) | 57 (30.8) | 0.01 |
| 7–8 hours, n (%) | 41 (10.5) | 13 (6.3) | 28 (15.1) |  |
| 8–9 hours, n (%) | 154 (39.3) | 79 (38.2) | 75 (40.5) |  |
| ≥ 9 hours, n (%) | 52 (13.3) | 27 (13.0) | 25 (13.5) |  |
| Falling asleep time,  h:mm, median (IQR) | 23:00 (22:30, 23:30) | 23:00 (22:30, 23:30) | 23:00 (22:00, 23:15) | 0.004 |
| Last waking up time,  h:mm, median (IQR) | 6:35 (6:30, 7:00) | 6:30 (6:30, 7:00) | 6:45 (6:30, 7:00) | 0.09 |
| Mid-sleep time, h:mm, median (IQR) | 2:45 (2:25, 3:05) | 2:45 (2:30, 3:07) | 2:45 (2:15, 3:00) | 0.09 |
| Weekend sleep habits |  |  |  |  |
| Sleep duration h, median (IQR) | 8.91 (8.00, 10.00) | 9.00 (8.00, 10.00) | 8.50 (8.00, 10.00) | 0.11 |
| < 8 hours, n (%) | 85 (21.7) | 42 (20.3) | 43 (23.2) | 0.20 |
| 8–9 hours, n (%) | 111 (28.3) | 54 (26.1) | 57 (30.8) |  |
| 9–10 hours, n (%) | 146 (37.2) | 78 (37.7) | 68 (36.8) |  |
| ≥ 10 hours, n (%) | 50 (12.8) | 33 (15.9) | 17 (9.2) |  |
| Falling asleep time,  h:mm, median (IQR) | 23:00 (22:40, 0:00) | 23:00 (23:00, 0:00) | 23:00 (22:30, 23:40) | 0.03 |
| Last waking up time,  h:mm, median (IQR) | 8:00 (7:20, 9:00) | 8:30 (7:30, 9:00) | 8:00 (7:00, 9:00) | 0.04 |
| Mid-sleep time, h:mm, median (IQR) | 3:45 (3:09, 4:30) | 3:45 (3:15, 4:30) | 3:30 (3:00, 4:15) | 0.005 |
| Corrected Mid-sleep time,  h:mm, median (IQR) | 3:15 (2:45, 3:51) | 3:17 (2:48, 3:53) | 3:10 (2:42, 3:51) | 0.14 |
| Chronotype |  |  |  |  |
| Morning type | 131 (33.4) | 64 (30.9) | 67 (36.2) | 0.53 |
| Intermediate type | 131 (33.4) | 71 (34.3) | 60 (32.4) |  |
| Evening type | 130 (33.2) | 72 (34.8) | 58 (31.4) |  |
| Weekly sleep loss, h, median (IQR) | 1.43 (0.71, 2.86) | 2.14 (1.43, 2.86) | 1.43 (0.71, 2.86) | 0.001 |
| < 1 hour, n (%) | 221 (56.4) | 100 (48.3) | 121 (65.4) | 0.001 |
| 1–2 hours, n (%) | 118 (30.1) | 69 (33.3) | 49 (26.5) |  |
| ≥ 2 hours, n (%) | 53 (13.5) | 38 (18.4) | 15 (8.1) |  |
| SJL, mm, median (IQR) | 60.0 (30.0, 95.62) | 65.0 (35.0, 97.5) | 52.5 (30.0, 90.0) | 0.03 |

AIS: Athens Insomnia Scale, IQR: Interquartile range, GAD: Generalized Anxiety Disorder, LSNS-6: Lubben Social Network Scale-6, PHQ: Patient Health Questionnaire, RLS: restless legs syndrome, RSQ: Restorative Sleep Questionnaire, SJL: social Jetlag.

Table S2. Grade differences demographic characteristics, psychosomatic symptoms, and sleep-wake characteristics

|  | Seventh grade | Eighth grade | Ninth grade | *P* value |
| --- | --- | --- | --- | --- |
| n | 122 | 143 | 127 |  |
| Male, n (%) | 52 (42.6) | 72 (50.3) | 61 (48.0) | 0.45 |
| Electronic devices user, n (%) | 69 (56.6) | 93 (65.0) | 95 (74.8) | 0.009 |
| Last electronic devices usage time |  |  |  |  |
| < 21:00, n (%) | 26 (21.3) | 19 (13.3) | 14 (11.0) | < 0.001 |
| 21:00–22:00, n (%) | 27 (22.1) | 31 (21.7) | 18 (14.2) |  |
| 22:00–23:00, n (%) | 46 (37.7) | 45 (31.5) | 37 (29.1) |  |
| ≥ 23:00, n (%) | 23 (18.9) | 48 (33.6) | 58 (45.7) |  |
| Headache, n (%) | 40 (32.8) | 49 (34.3) | 45 (64.3) | < 0.001 |
| ≥ 2 days/week, n (%) | 16 (13.1) | 19 (13.3) | 16 (12.6) | 1 |
| Abdominal pain, n (%) | 48 (39.3) | 49 (34.3) | 40 (31.5) | 0.43 |
| ≥ 2 days/week, n (%) | 19 (15.6) | 18 (12.6) | 11 (8.7) | 0.25 |
| Appetite loss, n (%) | 20 (16.4) | 31 (21.7) | 27 (21.3) | 0.52 |
| ≥ 2 days/week, n (%) | 9 (7.4) | 11 (7.7) | 11 (8.7) | 0.95 |
| Fatiguability, n (%) | 75 (61.5) | 89 (62.2) | 87 (68.5) | 0.44 |
| ≥ 2 days/week, n (%) | 52 (42.6) | 39 (27.3) | 53 (41.7) | 0.01 |
| Past history of COVID-19, n (%) | 95 (77.9) | 107 (74.8) | 94 (74.0) | 0.76 |
| COVID-19 unvaccinated person, n (%) | 76 (62.3) | 74 (51.7) | 45 (35.4) | < 0.001 |
| PHQ-9 score, median (IQR) | 2.0 (0.0, 5.0) | 1.0 (0.0, 5.0) | 2.0 (1.0, 5.5) | 0.03 |
| PHQ-9 score ≥ 5, n (%) | 32 (26.2) | 39 (27.3) | 41 (32.3) | 0.54 |
| GAD-7 score, median (IQR) | 3.0 (1.00, 5.75) | 1.0 (0.0, 3.0) | 1.0 (0.0, 2.0) | < 0.001 |
| GAD-7 score ≥ 5, n (%) | 40 (32.8) | 25 (17.5) | 9 (7.1) | < 0.001 |
| Loneliness scale score, median (IQR) | 4.0 (3.00, 5.00) | 3.0 (3.0, 4.0) | 3.0 (3.0, 3.0) | < 0.001 |
| Loneliness scale score ≥ 6, n (%) | 22 (18.0) | 7 (4.9) | 5 (3.9) | < 0.001 |
| LSNS-6 score, median (IQR) | 19.0 (14.0, 24.0) | 21.0 (16.0, 24.0) | 23.0 (19.0, 26.5) | < 0.001 |
| LSNS-6 score ≥ 12, n (%) | 21 (17.2) | 14 (9.8) | 5 (3.9) | 0.002 |
| AIS score, median (IQR) | 3.0 (2.0, 4.8 | 2.0 (1.0, 4.0) | 3.0 (2.0, 5.0) | 0.003 |
| AIS score ≥ 6, n (%) | 22 (18.0) | 16 (11.2) | 28 (22.0) | 0.05 |
| Difficulty initiating sleep, n (%) | 68 (55.7) | 64 (44.8) | 53 (41.7) | 0.07 |
| Moderate–severe, n (%) | 9 (7.4) | 13 (9.1) | 17 (13.4) | 0.28 |
| Difficulty maintaining sleep, n (%) | 21 (17.2) | 7 (4.9) | 16 (12.6) | 0.004 |
| Moderate–severe, n (%) | 2 (1.6) | 0 (0.0) | 1 (0.8) | 0.21 |
| Early wakening, n (%) | 19 (15.6) | 17 (11.9) | 17 (13.4) | 0.68 |
| Moderate–severe, n (%) | 4 (3.3) | 4 (2.8) | 2 (1.6) | 0.73 |
| Poor sleep quality, n (%) | 49 (40.2) | 42 (29.4) | 59 (46.5) | 0.01 |
| Moderate–severe, n (%) | 5 (4.1) | 2 (1.4) | 7 (5.5) | 0.17 |
| Daytime sleepiness, n (%) | 84 (68.9) | 96 (67.1) | 105 (82.7) | 0.007 |
| Moderate–severe, n (%) | 23 (18.9) | 19 (13.3) | 26 (20.5) | 0.26 |
| RSQ score, median (IQR) | 56.3 (43.8, 74.2) | 56.3 (42.2, 75.0) | 56.3 (40.6, 67.2) | 0.14 |
| RSQ score < 51.6, n (%) | 43 (35.2) | 58 (40.6) | 56 (44.1) | 0.37 |
| Difficulty getting up, n (%) | 27 (22.1) | 34 (23.8) | 31 (24.4) | 0.91 |
| RLS symptoms, n (%) | 18 (14.8) | 21 (14.7) | 23 (18.1) | 0.70 |
| ≥ 2 days/week, n (%) | 10 (8.2) | 7 (4.9) | 13 (10.2) | 0.24 |
| Average sleep duration h, median (IQR) | 8.29 (7.69, 8.79) | 8.00 (7.46, 8.61) | 7.57 (7.11, 8.25) | < 0.001 |
| < 7 hours, n (%) | 10 (8.2) | 22 (15.4) | 30 (23.6) | < 0.001 |
| 7–8 hours, n (%) | 33 (27.0) | 43 (30.1) | 51 (40.2) |  |
| 8–9 hours, n (%) | 57 (46.7) | 55 (38.5) | 39 (30.7) |  |
| ≥ 9 hours, n (%) | 22 (18.0) | 23 (16.1) | 7 (5.5) |  |
| Ideal sleep duration h, median (IQR) | 9.00 (8.00, 10.00) | 9.00 (8.00, 10.00) | 8.50 (8.00, 10.00) | 0.19 |
| Weekday sleep habits |  |  |  |  |
| Sleep duration h, median (IQR) | 8.00 (7.00, 8.50) | 8.00 (7.00, 8.50) | 7.00 (6.58, 8.00) | < 0.001 |
| < 7 hours, n (%) | 33 (27.0) | 47 (32.9) | 65 (51.2) | < 0.001 |
| 7–8 hours, n (%) | 12 (9.8) | 12 (8.4) | 17 (13.4) |  |
| 8–9 hours, n (%) | 56 (45.9) | 58 (40.6) | 40 (31.5) |  |
| ≥ 9 hours, n (%) | 21 (17.2) | 26 (18.2) | 5 (3.9) |  |
| Falling asleep time,  h:mm, median (IQR) | 22:30 (22:00, 23:00) | 23:00 (22:10, 23:30) | 23:00 (22:45, 0:00) | < 0.001 |
| Last waking up time,  h:mm, median (IQR) | 6:37 (6:30, 7:00) | 6:30 (6:30, 7:00) | 6:37 (6:30, 7:00) | 0.60 |
| Mid-sleep time, h:mm, median (IQR) | 2:30 (2:17, 3:00) | 2:45 (2:22, 3:00) | 3:00 (2:30, 3:21) | < 0.001 |
| Weekend sleep habits |  |  |  |  |
| Sleep duration h, median (IQR) | 9.00 (8.00, 10.00) | 8.67 (8.00, 10.00) | 8.00 (7.88, 9.00) | 0.005 |
| < 8 hours, n (%) | 22 (18.0) | 31 (21.7) | 32 (25.2) | 0.10 |
| 8–9 hours, n (%) | 26 (21.3) | 41 (28.7) | 44 (34.6) |  |
| 9–10 hours, n (%) | 54 (44.3) | 54 (37.8) | 38 (29.9) |  |
| ≥ 10 hours, n (%) | 20 (16.4) | 17 (11.9) | 13 (10.2) |  |
| Falling asleep time,  h:mm, median (IQR) | 23:00 (22:22, 23:30) | 23:00 (23:00, 0:00) | 23:10 (23:00, 0:00)  (*continued*) | < 0.001 |
| Last waking up time,  h:mm, median (IQR) | 8:00 (7:30, 9:00) | 8:00 (7:00, 9:00) | 8:00 (7:27, 9:00) | 0.97 |
| Mid-sleep time, h:mm, median (IQR) | 3:30 (3:00, 4:00) | 3:45 (3:15, 4:30) | 3:45 (3:15, 4:30) | 0.04 |
| Corrected Mid-sleep time,  h:mm, median (IQR) | 3:04 (2:33, 3:38) | 3:21 (2:53, 3:58) | 3:15 (2:53, 3:55) | 0.01 |
| Chronotype |  |  |  |  |
| Morning type | 54 (44.3) | 39 (27.3) | 38 (29.9) | 0.04 |
| Intermediate type | 36 (29.5) | 50 (35.0) | 45 (35.4) |  |
| Evening type | 32 (26.2) | 54 (37.8) | 44 (34.6) |  |
| Weekly sleep loss, h, median (IQR) | 1.43 (1.43, 2.86) | 1.43 (0.71, 2.86) | 1.43 (0.71, 2.86) | 0.87 |
| < 1 hour, n (%) | 65 (53.3) | 80 (55.9) | 76 (59.8) | 0.20 |
| 1–2 hours, n (%) | 42 (34.4) | 47 (32.9) | 29 (22.8) |  |
| ≥ 2 hours, n (%) | 15 (12.3) | 16 (11.2) | 22 (17.3) |  |
| SJL, mm, median (IQR) | 60.0 (36.3, 86.3) | 60.0 (30.0, 105.0) | 52.5 (30.0, 90.0) | 0.32 |

AIS: Athens Insomnia Scale, IQR: Interquartile range, GAD: Generalized Anxiety Disorder, LSNS-6: Lubben Social Network Scale-6, PHQ: Patient Health Questionnaire, RLS: restless legs syndrome, RSQ: Restorative Sleep Questionnaire, SJL: social Jetlag.

Table S3. Comparisons of demographic and psychosomatic symptoms between PHQ-9 score < 5 group and PHQ-9 score ≥ 5 group

|  | PHQ-9 < 5 group | PHQ-9 ≥ 5 group | *P* value |
| --- | --- | --- | --- |
| n | 280 | 112 |  |
| Male, n (%) | 145 (51.8) | 40 (35.7) | 0.005 |
| Grade |  |  |  |
| seventh, n (%) | 90 (32.1) | 32 (28.6) | 0.54 |
| eighth, n (%) | 104 (37.1) | 39 (34.8) |  |
| ninth, n (%) | 86 (30.7) | 41 (36.6) |  |
| Electronic devices user, n (%) | 186 (66.4) | 71 (63.4) | 0.64 |
| Last electronic devices usage time |  |  |  |
| < 21:00, n (%) | 40 (14.3) | 19 (17.0) | 0.11 |
| 21:00–22:00, n (%) | 54 (19.3) | 22 (19.6) |  |
| 22:00–23:00, n (%) | 101 (36.1) | 27 (24.1) |  |
| ≥ 23:00, n (%) | 85 (30.4) | 44 (39.3) |  |
| Headache, n (%) | 73 (30.7) | 61 (62.9) | < 0.001 |
| ≥ 2 days/week, n (%) | 16 (5.7) | 35 (31.2) | < 0.001 |
| Abdominal pain, n (%) | 76 (27.1) | 61 (54.5) | < 0.001 |
| ≥ 2 days/week, n (%) | 23 (8.2) | 25 (22.3) | < 0.001 |
| Appetite loss, n (%) | 31 (11.1) | 47 (42.0) | < 0.001 |
| ≥ 2 days/week, n (%) | 7 (2.5) | 24 (21.4) | < 0.001 |
| Fatiguability, n (%) | 153 (54.6) | 98 (87.5) | < 0.001 |
| ≥ 2 days/week, n (%) | 70 (25.0) | 74 (66.1) | < 0.001 |
| Past history of COVID-19, n (%) | 210 (75.0) | 86 (76.8) | 0.80 |
| COVID-19 unvaccinated person, n (%) | 142 (50.7) | 53 (47.3) | 0.93 |
| PHQ-9 score, median (IQR) | 1.0 (0.0, 2.0) | 7.0 (6.0, 10.0) | < 0.001 |
| GAD-7 score, median (IQR) | 1.0 (0.0, 3.0) | 1.0 (0.0, 4.0) | 0.71 |
| GAD-7 score ≥ 5, n (%) | 52 (18.6) | 22 (19.6) | 0.89 |
| Loneliness scale score, median (IQR) | 3.0 (3.0, 4.0) | 3.0 (3.0, 4.0) | 0.48 |
| Loneliness scale score ≥ 6, n (%) | 25 (8.9) | 9 (8.0) | 0.85 |
| LSNS-6 score, median (IQR) | 21.0 (16.0, 25.0) | 21.0 (16.0, 25.0) | 0.93 |
| LSNS-6 score ≥ 12, n (%) | 29 (10.4) | 11 (9.8) | 1 |

IQR: Interquartile range, GAD: Generalized Anxiety Disorder, LSNS-6: Lubben Social Network Scale-6, NRS: nonrestorative sleep, PHQ: Patient Health Questionnaire, RS: Restorative sleep, RSQ: Restorative Sleep Questionnaire.

Table S4. Comparisons of sleep characteristics between PHQ-9 score < 5 group and PHQ-9 score ≥ 5 group

|  | PHQ-9 < 5 group | PHQ-9 ≥ 5 group | *P* value |
| --- | --- | --- | --- |
| n | 280 | 112 |  |
| AIS score, median (IQR) | 2.0 (1.0, 4.0) | 4.0 (2.0, 6.3) | < 0.001 |
| AIS score ≥ 6, n (%) | 29 (10.4) | 37 (33.0) | < 0.001 |
| RSQ score, median (IQR) | 62.5 (50.0, 75.0) | 43.8 (34.4, 56.3) | < 0.001 |
| RSQ score < 51.6, n (%) | 84 (30.0) | 73 (65.2) | < 0.001 |
| Difficulty getting up, n (%) | 51 (18.2) | 41 (36.6) | < 0.001 |
| RLS symptoms, n (%) | 30 (10.7) | 32 (28.6) | < 0.001 |
| ≥ 2 days/week, n (%) | 15 (5.4) | 15 (13.4) | 0.01 |
| Average sleep duration h, median (IQR) | 8.00 (7.50, 8.57) | 7.71 (7.05, 8.29) | 0.001 |
| < 7 hours, n (%) | 35 (12.5) | 27 (24.1) | 0.004 |
| 7–8 hours, n (%) | 85 (30.4) | 42 (37.5) |  |
| 8–9 hours, n (%) | 120 (42.9) | 31 (27.7) |  |
| ≥ 9 hours, n (%) | 40 (14.3) | 12 (10.7) |  |
| Ideal sleep duration h, median (IQR) | 9.00 (8.00, 9.50) | 9.00 (8.00, 10.00) | 0.03 |
| Weekday sleep habits |  |  |  |
| Sleep duration h, median (IQR) | 8.00 (7.00, 8.00) | 7.25 (6.50, 8.00) | 0.001 |
| Falling asleep time, h:mm, median (IQR) | 23:00 (22:00, 23:30) | 23:00 (22:30, 23:30) | 0.001 |
| Last waking up time, h:mm, median (IQR) | 6:40 (6:30, 7:00) | 6:30 (6:23, 7:00) | 0.65 |
| Weekend sleep habits |  |  |  |
| Sleep duration h, median (IQR) | 9.00 (8.00, 10.00) | 8.50 (7.50, 10.00) | 0.11 |
| Falling asleep time, h:mm, median (IQR) | 23:00 (22:30, 23:30) | 23:30 (23:00, 0:30) | 0.002 |
| Last waking up time, h:mm, median (IQR) | 8:00 (7:00, 9:00) | 8:30 (7:30, 9:31) | 0.11 |
| Corrected mid-sleep time, h:mm, median (IQR) | 3:13 (2:45, 3:45) | 3:24 (2:46, 4:08) | 0.06 |
| Chronotype |  |  |  |
| Morning type | 97 (34.6) | 34 (30.4) | 0.11 |
| Intermediate type | 99 (35.4) | 32 (28.6) |  |
| Evening type | 84 (30.0) | 46 (41.1) |  |
| Weekly sleep loss, h, median (IQR) | 1.43 (0.71, 2.86) | 1.43 (0.71, 2.86) | 0.16 |
| < 1 hour, n (%) | 163 (58.2) | 58 (51.8) | 0.16 |
| 1–2 hours, n (%) | 85 (30.4) | 33 (29.5) |  |
| ≥ 2 hours, n (%) | 32 (11.4) | 21 (18.8) |  |
| SJL, mm, median (IQR) | 60.0 (30.0, 90.0) | 60.0 (30.0, 106.3) | 0.37 |

AIS: Athens Insomnia Scale, IQR: Interquartile range, RLS: restless legs syndrome, RSQ: Restorative Sleep Questionnaire, SJL: social Jetlag.

Figure S1. Histogram of RSQ score

RSQ_score

frequency

0

20

40

60

80

100

0

10

20

30

40

50

60

The histogram of RSQ score with bins of eight shows non-normal distribution. Although not clearly bimodal, two peaks are observed at 40-48 and 56-64. If a cut-off value of RSQ score is to be set, it should be somewhere in between these two peaks. RSQ: Restorative Sleep Questionnaire.

Figure S2. ROC curve of RSQ score for PHQ-9 ≥ 5

Specificity

Sensitivity

1.0

0.8

0.6

0.4

0.2

0.0

0.0

0.2

0.4

0.6

0.8

1.0

53.1 (0.65, 0.71)

Receiver operating characteristic (ROC) curve analysis was used to derive cut-off value of RSQ score for PHQ-9 ≥ 5. The RSQ score with the maximum sum of sensitivity and specificity was 53.1, with a sensitivity of 0.65, a specificity of 0.71, and an AUC (95% CI) of 0.73 (0.68, 0.79). CI: confidence interval, PHQ: Patient Health Questionnaire, ROC; receiver operating characteristic, RSQ: Restorative Sleep Questionnaire.

Figure S3. ROC curve of RSQ score for AIS ≥ 6

Specificity

Sensitivity

1.0

0.8

0.6

0.4

0.2

0.0

0.0

0.2

0.4

0.6

0.8

1.0

50.0 (0.65, 0.65)

Receiver operating characteristic (ROC) curve analysis was used to derive cut-off value of RSQ score for AIS ≥ 6. The RSQ score with the maximum sum of sensitivity and specificity was 50.0, with a sensitivity of 0.65, a specificity of 0.65, and an AUC (95% CI) of 0.70 (0.63, 0.77). AIS: Athens Insomnia Scale, CI: confidence interval, ROC; receiver operating characteristic, RSQ: Restorative Sleep Questionnaire.
